# Obituary

**Published:** 1885-08

**Authors:** 


### EDITORIAL.

ALABAMA, May, 1885.

DEAR DR.--Do you hold to the same views now on the use of the file that you did when you wrote your work on operative dentistry? Do you teach its use and benefits in the College? Can the file be well dispensed with in your practice? Some members of the Southern Dental Association contend that the file is going into disuse in the colleges, and that it is an old time practice. I cannot think so. Will be pleased to have your opinion. Respectfully, S. G. R.

In regard to the use of the file in dental operations, I have only changed views and opinion so far as it became necessary by the introduction of other instruments to take its place. The disks, the corrundum, but more especially the diamond disk, in the hands of the skillful operator, will better and more rapidly accomplish much that was formerly done with the file. There are still, however, many some in which these are not applicable and the file must be used.

There are many operators, in whose hands the disk is a dangerous instrument; such should confine themselves mainly to the use of the file.

The proper use of the chisel or heavy cutting instrument renders the use of the file less frequent than formerly.

I presume that the use of the file in dental operations will never be be wholly superseded; still it is not safe to affirm what will be in the future, even in this matter. The quality of files made for operations upon the teeth, is certainly relatively if not absolutely much inferior to those made twenty years ago.

Efforts in the improvement of the file have not been exercised as in the manufacture of other appliances, because of the impression that the file is becoming a thing of the past. This is unfortunate, many have become discouraged in the use of files upon the teeth, and especially the thinner varieties, because of their inferiority; some tempered to the friability of glass, others so soft as to become smooth at the first touch of enamel or dentine.

Let the file be used when and where it is best; make the most of it while it remains, and when the work for which it is used can be better done by something else, then it will be regulated to the list of obsolete things.

### Obituary.

Died, July 21st, 1885, in Mount Sterling, Ky., Dr. Richard M. Adair. Dr. Adair has been a prominent and successful dentist in Central Kentucky for many years. He graduated in the Baltimore College of Dental Surgery in 1857, and practiced his profession for almost the entire time, up to his death. His practice was confined mainly to Paris and Mount Sterling. His wife and two children survive him. He was a genial, courteous, christian gentleman, kind of heart, and devotedly attached to his friends. His wife and children have the sincere sympathy of all who knew him.

Died, July 15, 1885, of old age, at his residence, 3223 Chestnut street, St. Louis, Isaiah Forbes, D D.S., aged 75 years.

At a meeting of the St. Louis Dental Society, held July 16, 1885, a committee, composed of Drs. Spaulding, Eames, and Bowman was appointed to prepare suitable resolutions on the death of Dr. Forbes.

The following resolutions were reported:

Whereas, Isaiah Forbes, D.D.S., an old and much esteemed member of this society, and one of its founders, having departed this life on the 15th of this month, and this society being desirous of giving formal expression to the high regard in which he has ever been held by the members, adopt the following resolutions:

*Resolved*, That in the death of Dr. Isaiah Forbes this society and the profession of dentistry have lost a worthy and valuable member, one who deserved and received the confidence and respect of his fellow practitioners, and who was an ornament to the profession to which he belonged.

*Resolved*, That these resolutions be spread upon the record of this society; that a copy of these be sent to the family of the deceased, and that a copy be furnished for publication.

C. W. SPALDING,
W. H. EAMES,
GEO. A. BOWMAN,
Committee.

Pending their consideration, Dr. Spalding made the following remarks, after which the resolutions were unanimously adopted.

*Dr. C. W. Spalding.*—It is with profound emotion that I rise to make a few remarks on this occasion. Another member of that united band who represented the dental profession of St. Louis in former years, and who achieved for the profession of our city an enviable name wherever the dentists of St. Louis were known, has passed away. With the exception of one, who was a much younger member of that company, I stand here tonight as the sole survivor of what I think may with propriety be called "the Old Guard." They guarded well the interests of the profession in this city, and, what is still more creditable, they guarded the good name and the professional standing of every member of that faithful band. What touched one, touched all, and intercourse between them was characterized by disinterested friendship, frank and open speech, even to the pointing out of individual faults, and the honest and conscientious discharge of their duties to each other in accordance with the usages of the highest ethics of professional men.

That our friend was conscientious in the discharge of his professional duties, we can all testify. That he was just and kind, as well as frank and generous, we all well know. That he filled prominent positions in other walks of life, is evident from the interest other associations besides this take in the occasion of his death.

It has been said that all men are born to die. This is true the physical body of man, but not of the man himself. At the period called death the physical body is laid aside as one would cast off a garment; and in this instance an old and much worn garment that refused longer to serve the purposes for which it was formed. What we call death is simply a change from one stage of existence to another. That our friend is not dead is evidenced by our assembling here to-night. 'Tis true, memory of the man has its influence in bringing us together, but the tie that binds—the thread that draws us together, is not mere memory of the individual, but rather our rememberance of his worth. The life of every man, his acts, his words, his works, comprise all that we know and remember of him; for it is the character of a man that impresses itself upon our memories. His attributes become incorporated into the current humanity of his generation, and constitute a part of the active living forces of that period. These never die, but exert an influence on all future generations. Not only this, but the individual man does not die. That which is called death is but the portal to life immortal. It is birth into a life more real, more substantial and therefore more enduring, higher, deeper, fuller, with vaster capabilities, nobler aspirations, and incomparably grander results than belong to this poor life we are now living.

How insignificant then is this life compared with that upon which our friend has just entered! How trivial are all its affairs, when contrasted with those of an unending existence! Our birth and death are but the first and second mile-stones that mark our progress in that long, long journey whose limits we can not measure and whose worth we cannot estimate. Let us not then squander our brief hour in a struggle for a mere handful of earthly goods; let us rather imitate our worthy friend who has gone before us by but a few short years, and emulate his example by striving to serve our race rather than ourselves, and thus, like him, leave behind an unblemished name and a cherished memory.

